# Recent Advances in Intraoral Scanners

**DOI:** 10.1177/00220345241271937

**Published:** 2024-10-09

**Authors:** F. Eggmann, M.B. Blatz

**Affiliations:** 1 Department of Periodontology, Endodontology, and Cariology, University Center for Dental Medicine Basel UZB, University of Basel, Basel, Switzerland; 2 Department of Preventive and Restorative Sciences, Robert Schattner Center, Penn Dental Medicine, University of Pennsylvania, Philadelphia, PA, USA

**Keywords:** computer-assisted image processing, dental impression technique, dental models, oral diagnosis, permanent dental restoration, prostheses and implants

## Abstract

Intraoral scanners (IOSs) have emerged as a cornerstone technology in digital dentistry. This article examines the recent advancements and multifaceted applications of IOSs, highlighting their benefits in patient care and addressing their current limitations. The IOS market has seen a competitive surge. Modern IOSs, featuring continuous image capture and advanced software for seamless image stitching, have made the scanning process more efficient. Patient comfort with IOS procedures is favorable, mitigating the discomfort associated with conventional impression taking. There has been a shift toward open data interfaces, notably enhancing interoperability. However, the integration of IOSs into large dental institutions is slow, facing challenges such as compatibility with existing health record systems and extensive data storage management. IOSs now extend beyond their use in computer-aided design and manufacturing, with software solutions transforming them into platforms for diagnostics, patient communication, and treatment planning. Several IOSs are equipped with tools for caries detection, employing fluorescence technologies or near-infrared imaging to identify carious lesions. IOSs facilitate quantitative monitoring of tooth wear and soft-tissue dimensions. For precise tooth segmentation in intraoral scans, essential for orthodontic applications, developers are leveraging innovative deep neural network–based approaches. The clinical performance of restorations fabricated based on intraoral scans has proven to be comparable to those obtained using conventional impressions, substantiating the reliability of IOSs in restorative dentistry. In oral and maxillofacial surgery, IOSs enhance airway safety during impression taking and aid in treating conditions such as cleft lip and palate, among other congenital craniofacial disorders, across diverse age groups. While IOSs have improved various aspects of dental care, ongoing enhancements in usability, diagnostic accuracy, and image segmentation are crucial to exploit the potential of this technology in optimizing patient care.

## Introduction

Intraoral scanners (IOSs) are integral to digital dentistry. They allow for the acquisition of intraoral optical impressions (IOIs) and the representation of data as detailed virtual models. Recent years have witnessed notable IOS technology advancements, characterized by hardware innovations and software breakthroughs. These developments improved intraoral scanning efficiency and accuracy in computer-aided design and computer-aided manufacturing (CAD-CAM), which remains IOSs’ most common use case.

**Figure 1.**
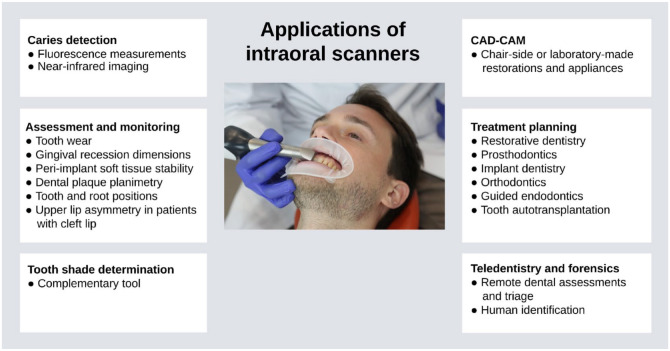
Schematic diagram with an overview of the diverse applications of contemporary intraoral scanners (IOSs).

In synergy with imaging methods such as cone-beam computed tomography (CBCT) and 3-dimensional facial scans, technological advancements of IOSs have also broadened their capabilities. IOS applications now extend beyond impression taking to include diagnostics, treatment planning, and monitoring (Fig. 1). With the swift pace of advancements in digital and imaging technologies, IOSs are poised to play an increasingly vital role across diverse dentistry and oral and maxillofacial surgery specialties.

This article explores IOS advancements and applications, highlighting the benefits they offer in patient care and addressing their current limitations. The initial sections provide an overview of the latest technological progress in IOS hardware and software and survey novel diagnostic applications of IOSs. The article then examines patient and user experiences and the accuracy of IOSs. Drawing from the most recent 3 y of published research, this article provides an overview of IOS advancements, rather than an exhaustive review of all IOS use cases.

Concluding with a forward-looking perspective, the article summarizes the current state of IOS technology and its potential future directions. Emphasis is placed on the challenges and the importance of continued research in fully leveraging IOS technology to enhance patient care.

## Advancements in IOS Technology

It is worth noting that detailed published data on hardware and software systems are scarce. While public and nonprofit research institutions are essential in advancing technological progress, the innovations in IOSs that are used in patient care are predominately driven by IOS manufacturers. These companies closely guard the technological specifics of their systems, limiting the availability of comprehensive information. Patent databases, such as those maintained by the United States Patent and Trademark Office and the European Patent Office, offer some insights into the innovations made by IOS manufacturers. However, patents provide neither an accurate nor a comprehensive picture of the technologies currently in use or those anticipated for future implementation.

### Hardware Developments

Key IOS advancements include faster scanning, no scanning powder, and color image acquisition, which enhance procedure efficiency, patient comfort, and user experience. Following these step changes, recent years have seen more incremental improvements in IOS hardware. Concurrently, the market for IOSs has become highly competitive, with established brands refining models and newcomers entering the market, emphasizing either affordable pricing or integrated software solutions as their unique selling propositions (Al-Hassiny et al. 2023).

The core design of IOS systems has largely stayed the same, but unique ergonomic features set each model apart, influencing user preferences. A trend is the shift from corded to wireless, battery-powered devices (Al-Hassiny et al. 2023). This shift offers increased mobility and convenience but raises concerns about reduced operational time and connectivity issues. Some models are also equipped with haptic feedback, offering guidance during image acquisition. Most IOS scanning heads have integrated heaters to mitigate condensation during extended use. IOS devices come as standalone scanners or as parts of integrated CAD-CAM platforms, facilitating chairside restoration fabrication (Bandiaky et al. 2022). An advancement is autocalibration in some IOS devices, enhancing user-friendliness by reducing regular manual calibration needs.

However, despite these advancements, IOS technology still faces challenges, including the necessity for a dry operating field to avoid measurement errors caused by oral fluids and difficulties in capturing subgingival preparation margins (Tabesh et al. 2022).

### Software Innovations

Software advancements have outpaced hardware in expanding IOS capabilities. Modern IOSs capture images continuously, with advanced algorithms stitching images seamlessly to optimize scanning. IOSs are capable of simultaneously capturing and processing the surface features and their optical interactions across a specified area, employing oversampling and averaging multiple measurement points representing the same area.

Advancements in software differentiate IOS manufacturers in a competitive market. Some IOSs are designed for scanning and data export, while others evolve into comprehensive platforms for more than just IOIs. These advanced systems offer access to a wide range of software applications that enable dental practitioners to enhance diagnostics, patient communication, monitoring, and treatment planning. Tailored software applications now cover various functions, including CAD-CAM, implant planning, smile design, and orthodontic simulation.

Moreover, specialized software enhancements are employed to eliminate extreme points, thereby minimizing noise and image artifacts. They also omit superfluous imaging data, ensuring extraneous elements like the buccal mucosa and tongue are excluded from IOIs.

The transition from closed systems restricting CAD-CAM to proprietary solutions to open interfaces has marked a major industry change. Of the 21 most used IOSs listed in a recent survey, all devices are equipped with an open interface (Al-Hassiny et al. 2023). This feature, enabling the export of IOI data in at least 1 standard file format, provides users with increased flexibility, better interoperability, and enhanced customization options.

Several file formats, including STL, OBJ, and PLY, store IOI data, each with unique advantages and limitations (Turkyilmaz et al. 2020). STL, which encodes IOIs as meshes of tessellated triangles, remains the most prevalent file format. In contrast, OBJ and PLY are file formats that employ polygonal facets, freeform curves, and freeform patches for data encoding. These formats offer better accuracy and store texture and color information. They facilitate easier model modification but require more storage space owing to the higher volume of data they accommodate.

#### Three-dimensional tooth segmentation

The utility of accurate IOSs extends far beyond mere image capture, relying on advanced software for in-depth analysis. Notably, precise segmentation of each individual tooth from IOI data is crucial in various applications such as diagnosis and treatment planning in orthodontics, orthognathic surgery, and prosthodontics. This segmentation allows clinicians to perform space analysis, treatment simulation, movement predictions, and tooth shape analysis (Hao et al. 2022).

However, tooth segmentation must account for significant variability among patients and ensure accurate boundary detection between teeth and gingiva (Hao et al. 2022). This precision is essential to provide reliable segmentation outputs, as even minor errors can have repercussions in treatment planning (Hao et al. 2022). Consequently, automatic tooth segmentation is challenging (Luo et al. 2021; Hao et al. 2022).

Tooth segmentation software has often relied heavily on high-quality labeled datasets, requiring substantial human effort for annotation. This limits dataset scale (Vinayahalingam et al. 2023). For example, a fully automated, fault-aware system based on deep learning achieved impressive results in tooth segmentations, with 94% of cases being deemed clinically viable by experts without the need for additional corrections (Hao et al. 2022). However, the system required training with 4,000 IOIs, each manually labeled by experts.

A novel approach using deep neural networks with unsupervised pretraining and supervised fine-tuning achieves precise tooth segmentation in IOIs (Liu et al. 2023). This method mitigates the reliance on extensive labeled training data, paving the way for more streamlined tooth segmentation processes and, subsequently, the advancement of diagnostic technologies.

#### Landmarking and model analysis

Manually placing landmarks on dental models, essential for diagnostics and outcome assessments in orthodontics, is error-prone and time-consuming. To address this, software has been developed that uses a combination of machine learning and linear programming to automatically recognize and label each tooth and its landmarks (Woodsend et al. 2021). While this approach still necessitates human verification, it facilitates the rapid and precise identification of landmarks on dental models. This represents a significant advancement toward the automation of routine clinical evaluations, such as the “Index of Orthodontic Treatment Need.”

However, it is important to note that while automated digital model analyses are highly reproducible, their results can differ from those obtained through manual measurements (Yu et al. 2023). This highlights a crucial aspect of these automated systems: they offer rapidity and consistency in results. Yet, for certain detailed assessments in which utmost precision is paramount, manual methods may still hold a clear advantage.

Software, underpinned by deep learning, can categorize molars in IOIs according to the presence and type of restoration present (Eto et al. 2022). This represents an initial advancement toward creating automated systems designed to produce dental charts directly from IOIs.

## Diagnostic Capabilities of IOSs

Technological advancements have not only enhanced the user experience but also expanded the diagnostic capabilities of IOSs. This is evident in the application of IOSs for caries detection, tooth wear monitoring, oral hygiene assessment, soft-tissue evaluation, and tooth shade determination.

### Caries Detection

IOS tools for detecting caries are based either on fluorescence technologies using light with a wavelength of 415 nm or on near-infrared imaging, which employs light wavelengths between 727 nm and 850 nm (Schlenz et al. 2022). Depending on the specifications set by their manufacturers, IOSs support detection of either proximal or occlusal carious lesions or, in some instances, both.

In a study conducted in clinical and laboratory settings, the performance of an IOS was compared with visual examination for identifying occlusal caries in permanent molars (Michou et al. 2021). Histological analysis after tooth extraction served as the reference standard. The study demonstrated that the diagnostic accuracy of the IOS, using fluorescence measurements, was commensurate with that of visual examinations. This finding was corroborated in a subsequent in vivo study using the same IOS (Ntovas et al. 2023). In clinical settings, the IOS exhibited negligible deviation in accuracy compared with laboratory conditions, which implies a consistent performance across different environments (Michou et al. 2021). However, the study also highlighted the risk of false-positive results due to the presence of biofilm on occlusal surfaces. This underscores the liability of IOS fluorescence assessments to external factors, which carry the risk of leading to diagnostic errors.

Further evidence comes from a clinical study with data from 100 patients, in which an IOS capturing color images and near-infrared imaging data simultaneously exhibited noninferiority to bitewing radiography, with the latter serving as reference standard (Metzger et al. 2022). Experts in assessing scans featuring near-infrared imaging attained a higher diagnostic accuracy than novices did, indicating a learning curve essential for optimizing the benefits of such features (Metzger et al. 2022).

Despite these promising findings, evidence on the diagnostic accuracy of IOSs featuring near-infrared imaging remains equivocal. A laboratory study found that this technology has a high sensitivity in detecting enamel lesions, advantageous for noninvasive caries management (Michou et al. 2022). In contrast, a clinical study showed that another IOS with near-infrared imaging has low sensitivity in pediatric patients, with the most reliable results achieved by combining IOIs with bitewing radiography (Cuenin et al. 2023).

Beyond caries detection, IOSs can aid in evaluating developmental enamel defects. However, the magnification and color enhancement features of IOSs entail a risk of overestimating the extent of defects such as dental fluorosis and molar-incisor hypomineralization (Cardoso-Silva et al. 2023).

In summary, the body of evidence underscores the potential of IOSs in caries detection but also points to the need for usability and accuracy enhancements. IOSs, when optimized, can serve as valuable adjuncts in caries detection, facilitating more reliable assessments. By decreasing the reliance on radiographic evaluation, IOSs can also contribute to reducing exposure to ionizing radiation for patients.

### Tooth Wear Monitoring

Some IOSs use image superimposition software based on best-fit alignment. This software enables quantitative assessment of surface alterations. In a controlled in vitro setting, these IOSs align closely with profilometric measurements, maintaining an accuracy margin of ±15 µm (Witecy et al. 2021). This level of precision sets them apart from semi-quantitative tooth wear indices.

However, the lack of stable oral reference points makes best-fit alignment prone to inaccuracies (Charalambous et al. 2022). While time-consuming strategies exist to mitigate this, such as manually selecting reference points or zones, there is a need for further software enhancements to improve scan registration accuracy.

In clinical settings, the repeatability error for IOS tooth wear analyses falls between 60 and 70 µm (Bronkhorst et al. 2023). A discrimination threshold of about 73 µm, beyond which measurements are likely to accurately represent tooth wear, was also determined for another IOS in a study using natural teeth (Charalambous et al. 2022). Together, these studies indicate that although the measurement technique provides low precision for detecting minute levels of wear, it can reliably discern and track changes surpassing the specified thresholds.

Given current accuracy constraints and typical tooth wear rates, periodic IOIs (every 1–3 y) are valuable for monitoring and guiding management strategies (Bronkhorst et al. 2023).

### Oral Hygiene Assessment

Planimetric methods enable quantification of dental plaque coverage, providing a detailed record of a patient’s or study participant’s oral hygiene status (Doi et al. 2021). A study involving 20 subjects illustrated the accuracy of plaque planimetry using imaging with an IOS after the application of a plaque disclosing agent (Jung et al. 2022). This approach facilitated a valid and efficient planimetric evaluation of all tooth surfaces across the dentition. It successfully addressed and overcame several limitations inherent to planimetry based on intraoral photographs, showcasing its potential as a superior method for meticulous oral hygiene assessment.

Moreover, a randomized controlled trial revealed improvements in periodontal and plaque parameters among patients who used a smartphone for intraoral scanning at home after nonsurgical treatment (Shen et al. 2022). In this study, patients received automated motivational messages that were based on machine learning–supported evaluations of visible supragingival plaque and signs of gingival inflammation. These findings underscore the potential of patient-conducted intraoral imaging via smartphones for monitoring and personalized oral health counseling, highlighting a promising avenue for enhancing oral hygiene management.

### Soft-Tissue Evaluation

IOSs are increasingly used to assess gingival recession and peri-implant soft-tissue stability (Kuralt et al. 2022; Kuralt and Fidler 2022). They are also used, with the aid of superimposition tools, to monitor soft-tissue changes over time (Dritsas et al. 2023; Song et al. 2023). Clinical and laboratory studies indicate that IOS assessments surpass conventional techniques in accuracy (Kuralt et al. 2022). However, IOS precision depends on the operator’s skill, the device, and the scan location (Kuralt et al. 2022). For example, acquiring accurate IOIs is fraught with challenges in interproximal and posterior areas.

### Tooth Shade Determination

Some IOSs with color imaging are used for tooth shade determination. While some investigations suggest that IOSs provide accuracy on par with visual shade selection, a systematic review found that current IOSs often fall short in shade-matching accuracy (Tabatabaian et al. 2022). Unlike spectrophotometers, which measure quantitative color values, IOSs equipped with color-matching tools yield only qualitative color data. The discrepancy in shade-matching accuracy between spectrophotometers and IOSs stems from IOSs’ inability to maintain uniform illumination (Tabatabaian et al. 2022). In addition, the angle of IOS color readings often deviates from the optimal 0° angle, causing further inaccuracies. Consequently, IOSs are recommended only as a supplementary tool for tooth shade determination (Akl et al. 2023).

## Clinical Integration

### Patient and User Experience: Benefits and Obstacles

Although the routine integration of IOS technologies for diagnostic purposes is still in development, their contribution to clinical patient care, particularly for CAD-CAM, is significant. Since IOSs eliminate some discomforts of conventional impression taking, patients rate procedure comfort more favorably (Bandiaky et al. 2022; Serrano-Velasco et al. 2023).

Survey data show a rise in dental practitioners either using IOSs or contemplating their adoption, predominantly for fabricating single-unit restorations (Revilla-Leon et al. 2021; Al-Hassiny et al. 2023). The main adoption driver for IOSs is improved clinical efficiency; nonusers cite cost as the biggest barrier.

Notwithstanding the advantages of IOSs, large dental institutions face obstacles in implementing IOSs. These challenges encompass integrating IOS software with current electronic health record systems, ensuring data security, managing data storage, and training clinicians and staff (Jahangiri et al. 2020). Thus, IOS adoption has been slower in high-volume institutions than in private practices (Jahangiri et al. 2020).

### IOS Accuracy: From Basic Scans to Complex Impressions

To be useful tools, IOSs need to be reliable and accurate. The accuracy of IOIs is measured by trueness and precision. Trueness refers to the degree of closeness of IOIs to the original object, while precision refers to the consistency of repeated scans. The root mean square error, a metric increasingly used for assessing accuracy, quantifies the differences between all corresponding points in the reference and experimental scans (Vitai et al. 2023). Lower root mean square error values indicate that the experimental scans closely align with the reference scan, denoting high accuracy. While IOS accuracy has generally improved, the latest IOSs do not always offer the highest accuracy (Vitai et al. 2023).

IOSs achieve clinically acceptable accuracy in scanning complete dentate arches (Vitai et al. 2023). Challenges persist, however, in edentulous areas. A systematic review incorporating data from 10 laboratory and 8 clinical studies found that that IOSs can match the accuracy of conventional impressions for recording denture-bearing soft tissues in cases in which the ridges are firm and have attached mucosa (Rasaie et al. 2021). Yet, in scenarios like complete denture fabrication or cases necessitating the recording of tissue movements, existing IOSs offer no suitable alternative to conventional methods, except for creating preliminary impressions.

**Figure 2.**
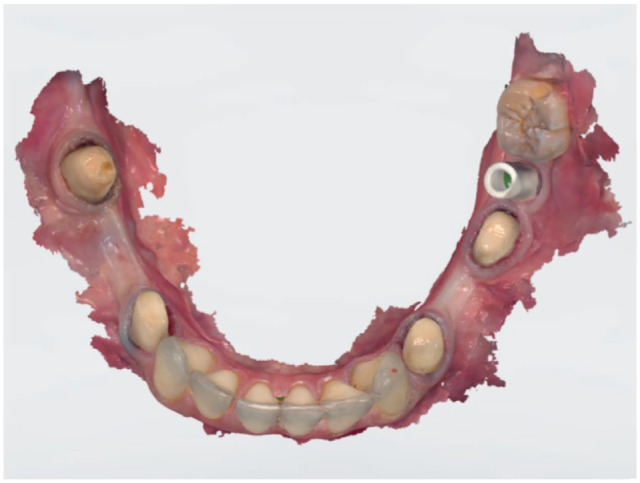
Intraoral optical impression of a mandibular arch with prepared teeth and an implant holding a scan body.

Dental implants, lacking the physiological mobility of teeth, necessitate heightened accuracy from IOSs for a passive fit of implant-supported restorations. A systematic review of 8 clinical studies has indicated that contemporary IOSs meet the accuracy requirements for digital impressions of single or multiple implants (Schmidt et al. 2022) (Fig. 2). However, their efficacy in capturing accurate full-arch impressions in implant cases is limited (Vitai et al. 2023).

To improve the accuracy of recording the positions of multiple implants, there has been a shift from vertically positioned scan bodies to horizontally positioned scan gauges. These gauges possess multiple planes on the top surface, reducing the need to rotate the IOS during scanning, which in turn results in less image stitching and fewer positional errors (Giglio et al. 2023).

**Figure 3.**
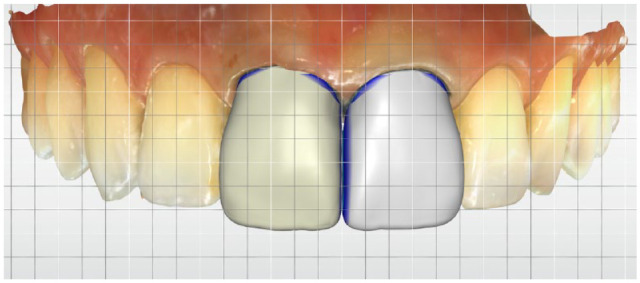
Digital design of monolithic crowns for maxillary central incisors using an intraoral scanner platform that includes copy-and-mirror tools to optimize the design process and a grid overlay to assist in precise alignment and proportion assessment.

A review of 12 in vitro and 6 in vivo studies found comparable marginal accuracy for crowns between IOS and conventional impressions; both approaches achieved acceptable results (Tabesh et al. 2022) (Fig. 3). This aligns with earlier findings from a review of 13 clinical studies on the marginal fit of crowns and 3-unit fixed partial prostheses (Bandiaky et al. 2022). Similarly, a systematic review of 8 randomized controlled trials revealed no significant difference in the clinical outcomes of fixed prostheses, whether tooth supported or implant supported, between IOS and conventional workflows (Mahat et al. 2023). However, it is crucial to recognize that substantial variations in accuracy exist—not only between different IOS technologies but also among different generations of a specific IOS.

### Digital Twins: Enhancing Treatment Planning

**Figure 4.**
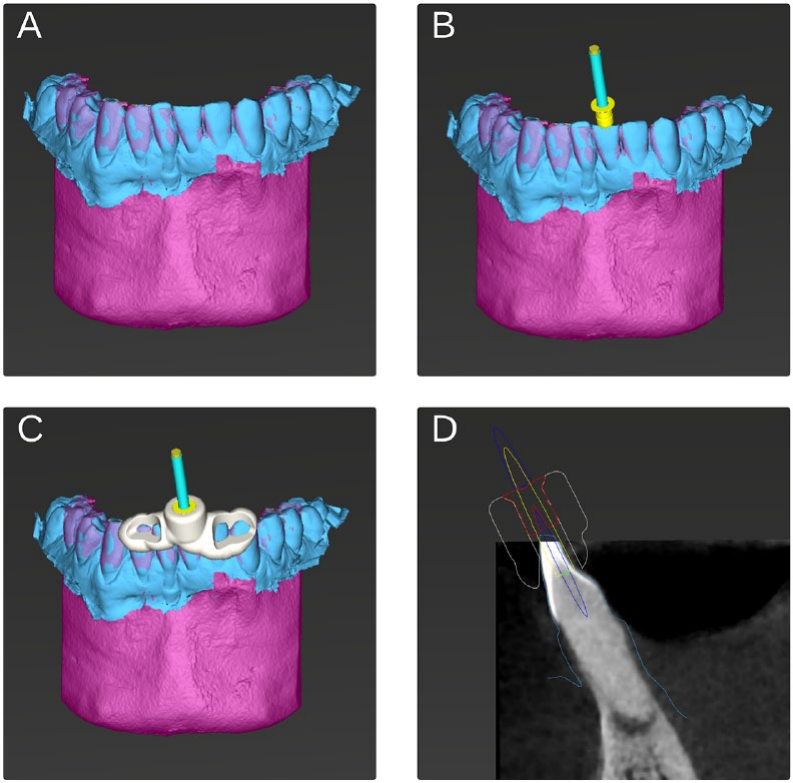
Digital workflow for guided endodontics using a digital twin model on an intraoral scanner platform. (**A–D**) Virtual treatment planning for an endodontic access cavity in a mandibular incisor affected by pulp canal calcification and apical periodontitis. (**A**) Registration of the intraoral optical impression, shown in blue, and the cone-beam computed tomography (CBCT) scan, represented in magenta. (**B**) Virtual planning of the access cavity based on CBCT imaging, ensuring optimal entry and minimal invasiveness, alongside the integration of a suitable sleeve system, designed to accurately guide the bur. (**C**) Planning of a custom guide that secures the sleeve system, incorporating inspection windows to verify the guide’s accurate placement and stability on the dental arch. (**D**) Sagittal view of the digital twin’s incisor, illustrating the detailed planning of the access cavity and the positioning of the bur, sleeve, and guide.

Integrated with CBCT or face scans, accurate IOSs are crucial in developing comprehensive treatment-planning platforms. This integration facilitates the creation of precise “digital twins”—3-dimensional (3D) models that provide accurate anatomical details and spatial relationships (Lee et al. 2023). These digital twins enhance the accuracy and efficacy of various dental procedures. For instance, IOI data combined with face scans enable digital smile design, while IOS scans merged with CBCT data support both static and dynamic navigation methods (Connert et al. 2022). These approaches are increasingly used in a range of procedures, including orthodontic mini-implant placement, preparation of endodontic access cavities (Fig. 4), insertion of dental implants, and tooth autotransplantation.

## Specialized and Emerging IOS Applications

### Innovations in Prosthodontics: Navigating Complexities with IOSs

In prosthodontics, accurately recording maxillomandibular relationships is crucial yet complex, especially in cases involving posterior free-end situations. In patients with a complete natural dentition, scanning the buccal surfaces of a quadrant, while teeth are in the maximum intercuspal position, provided higher accuracy in static interocclusal registration compared with full-arch scanning and physical bite recording with polyvinyl siloxane (Morsy and El Kateb 2022). In the context of multispan implant restorations, by contrast, digital bite registration continues to pose significant challenges (Joda et al. 2021).

The goal is to develop virtual articulators from patient-specific data, using IOSs for dynamic mandibular movement analysis. However, even with improved bite alignment of digital scans, minor translational inaccuracies in mesh alignments persist. These inaccuracies cause significant deviations in intercuspal relations, limiting clinical use (Safrany-Fark et al. 2023).

Given the persistent challenges of obtaining accurate complete-arch IOIs of implants, alternative methods such as photogrammetry are garnering attention. Photogrammetry involves capturing high-resolution photographs from various angles of implants, using screw-retained fiducial markers as references. This method uses advanced software to identify common points across photographs to construct accurate 3D models of dental implant positions. Capturing adjacent teeth and soft tissues requires an additional impression, via an IOI or conventional methods. A systematic review, encompassing data from 3 clinical trials, 6 laboratory studies, and 6 case reports, suggests that photogrammetry may serve as a reliable alternative for obtaining implant scans (Gómez-Polo et al. 2023). However, it is important to note that the evidence substantiating this is limited and primarily stems from studies focusing on implants in fully edentulous arches.

### Orthodontics: The Digital Shift

**Figure 5.**
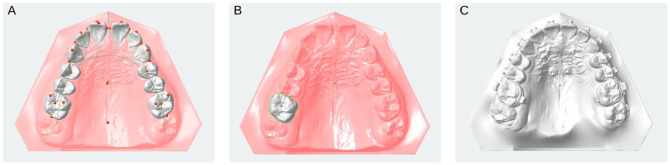
Digital orthodontic planning using an intraoral scanner platform. Panels A–C demonstrate orthodontic applications of an IOS platform. (**A**) Maxillary dental arch with landmark points marked on each tooth, facilitating detailed model analysis and treatment strategy formulation. While landmarks are initially identified automatically, manual adjustments may be required to refine their accuracy. (**B**) Focused view of a first molar after digital tooth segmentation. (**C**) Intraoral optical impression of a dental arch featuring a multibracket fixed orthodontic appliance and an orthodontic palatal implant, whose position can be determined without a scan body.

Adoption of orthodontic aligners for treating various malocclusions has increased. Concurrently, IOIs and virtual treatment planning have become practicable alternatives to conventional workflows (Fig. 5). However, evidence on the accuracy of orthodontic measurements from digital methods versus conventional casts is conflicting (Christopoulou et al. 2022). These discrepancies appear to vary based on the specific type of IOS used.

Orthodontics often requires full-arch scans with crowded teeth and appliances, posing unique challenges for digital impressions. Despite these challenges, a study involving 20 adolescents demonstrated that accurate digital models can be obtained from IOS scans taken with multibracket fixed orthodontic appliances in situ (Palone et al. 2023). This suggests that removing brackets and archwires for imaging may be unnecessary.

Besides diagnostics and treatment planning, IOSs can be used for monitoring root position. Merging CBCT datasets with pretreatment IOS data can generate detailed 3D tooth models. These models, integrated into digital scans taken during or after treatment, then permit accurate prediction of root position, obviating the need for additional CBCT imaging (Lee et al. 2022).

### Oral and Maxillofacial Surgery: Expanding IOS Utility

Like in orthodontics, IOSs find varied applications in oral and maxillofacial surgery. Retrospective studies from 3 cleft care centers demonstrated the successful application of IOSs across various age groups—from neonates to preschoolers (Benitez et al. 2022; Weise et al. 2022). These studies included individuals with cleft lip and palate as well as other congenital craniofacial disorders, demonstrating successful IOS use in patients whether awake or under general anesthesia. IOSs are versatile for diagnostics, treatment planning, and orthopedic appliance fabrication. For instance, IOSs can be used to fabricate appliances for nasoalveolar molding for infants and speech-enhancing obturator protheses in children with cleft lip and palate deformities (Shanbhag et al. 2020; Abreu et al. 2022).

Beyond improving airway safety during impression taking, IOSs streamline workflows and allow the integration of additional digital technologies, such as the automated design of presurgical orthopedic plates and quantitative assessment of upper lip scarring and asymmetry (Ayoub et al. 2021; Zarean et al. 2022; Schnabel et al. 2023).

Besides care for patients with cleft lip and palate, laboratory studies have illustrated that IOSs can digitize facial defects to clinically acceptable levels, potentially rivaling the scanning accuracy of extraoral optical systems (Dohiem et al. 2022; Unkovskiy et al. 2022). Considering that IOSs are more accessible and less expensive that extraoral facial scanners, employing IOSs emerges as a promising avenue for innovative digital solutions and cost reductions in maxillofacial prosthetics. However, it is crucial to note that the capabilities of IOSs have inherent limitations: certain defects, such as orbital defects, surpass their scope of applicability (Unkovskiy et al. 2022).

### Teledentistry: IOSs for Remote Care

IOSs enable data sharing among dental professionals, facilitating remote assessments. The findings of an observational diagnostic accuracy study suggest that remote assessments based on approximate true-color IOIs can be effective in detecting dental findings (Steinmeier et al. 2020). However, their accuracy in evaluating periodontal conditions was inconsistent (Steinmeier et al. 2020). Improved image quality and integration with supplementary patient data, such as radiographs, could potentiate IOSs as efficient tools for patient screening and triage in teledentistry.

Using smartphones for patient-conducted intraoral imaging presents potential benefits for identifying orthodontic aligner unseats and determining optimal timing for aligner changes. However, a retrospective study found that the evaluated system was unsuitable for use by itself, emphasizing the ongoing need for in-person monitoring and technological improvements (Ferlito et al. 2023).

### Forensic Dentistry: A New Frontier for IOSs

As in teledentistry, IOSs are also finding novel ground in forensic dentistry. Dental records are crucial for forensic identification, with anterior palate morphology helping to distinguish individuals (Simon et al. 2022; Mikolicz et al. 2023). Leveraging IOS imaging can speed up and refine the identification process if preexisting imaging data are available (Mikolicz et al. 2023).

## Conclusions and Perspective

IOSs are pivotal in advancing digital dentistry, offering multifaceted applications and substantial benefits in patient care. From CAD-CAM integration to treatment planning, diagnostics, and monitoring, IOSs enhance patient comfort and streamline various clinical workflows.

Several IOS applications stand out for their effectiveness and widespread adoption, notably in CAD-CAM integration for fabricating indirect restorations and orthodontics for treatment planning and appliance fabrication. Their capability of integrating with various imaging modalities for comprehensive planning underscores their role in delivering personalized care.

Machine learning, particularly deep learning, is currently used for image segmentation and possesses enormous potential to enhance the functionalities of IOSs. However, despite advancements, challenges remain. These encompass the incorporation of IOSs in large dental institutions, the accuracy in scanning edentulous areas and implants in cases with full-arch edentulism, and the need for improvements in diagnostic accuracy.

Future research and development are set to address several key areas. Evaluating the cost-effectiveness of IOSs in various dental settings is essential for guiding investment and implementation strategies. Refining user-friendly IOS software for seamless integration with existing dental systems is crucial for improving efficiency and usability. Further development is necessary to maximize IOSs’ potential as diagnostic tools and comprehensive platforms for individualized dental charts, treatment planning, and oral health monitoring. Increasing interest in IOSs for teledentistry, remote diagnosis, and care underscores their growing significance. In addition, using smartphones for patient-conducted intraoral imaging to enhance monitoring and personalized counseling is a promising area of exploration. Integrating IOSs with advanced imaging methods for virtual or augmented reality applications and robot-assisted procedures also represents a compelling research frontier.

Ongoing IOS technology developments highlight their potential to elevate dental care standards. Continued research and innovation are crucial to overcoming current limitations and fully realizing the potential of IOS technology in optimizing patient care.

## Author Contributions

F. Eggmann, M.B. Blatz, contributed to conception and design, data acquisition, analysis, and interpretation, drafted and critically revised the manuscript. Both authors gave their final approval and agree to be accountable for all aspects of the work.
